# Identification of Potentially Pathogenic Variants in the Posterior Polymorphous Corneal Dystrophy 1 Locus

**DOI:** 10.1371/journal.pone.0158467

**Published:** 2016-06-29

**Authors:** Derek J. Le, Duk-Won D. Chung, Ricardo F. Frausto, Michelle J. Kim, Anthony J. Aldave

**Affiliations:** Stein Eye Institute, David Geffen School of Medicine at UCLA, Los Angeles, California, United States of America; CNR, ITALY

## Abstract

Posterior polymorphous corneal dystrophy 1 (PPCD1) is a genetic disorder that affects corneal endothelial cell function and leads to loss of visual acuity. PPCD1 has been linked to a locus on chromosome 20 in multiple families; however, Sanger sequencing of protein-coding genes in the consensus region failed to identify any causative missense mutations. In this study, custom capture probes were utilized for targeted next-generation sequencing of the linked region in a previously reported family with PPCD1. Variants were detected through two bioinformatics pipelines and filtered according to multiple criteria. Additionally, a high-resolution microarray was used to detect copy number variations. No non-synonymous variants in the protein-coding region of annotated genes were identified. However, 12 single nucleotide variants in 10 genes, and 9 indels in 7 genes met the filtering criteria and were considered candidate variants for PPCD1. Eleven single nucleotide variants were confirmed by Sanger sequencing, including 2 synonymous variants and 9 non-coding variants, in 9 genes. One microdeletion was detected in an intron of *OVOL2* by microarray but was subsequently not identified by PCR. Using a comprehensive next-generation sequencing approach, a total of 16 genes containing single nucleotide variants or indels that segregated with the affected phenotype in an affected family previously mapped to the PPCD1 locus were identified. Screening of these candidate genes in other families previously mapped to the PPCD1 locus will likely result in the identification of the genetic basis of PPCD1.

## Introduction

The corneal dystrophies are a heterogeneous group of genetic disorders that are associated with bilateral, progressive loss of visual acuity due to changes in the cornea [1]. Four corneal dystrophies, posterior polymorphous corneal dystrophy (PPCD), Fuchs endothelial corneal dystrophy, congenital hereditary endothelial dystrophy, and X-linked endothelial corneal dystrophy, affect the corneal endothelium and are collectively known as the endothelial corneal dystrophies.

PPCD is characterized by bands, vesicles, and gray opacities at the level of the corneal endothelium and is associated with corneal steepening [2]. Extracorneal manifestations such as glaucoma, keratoconus, and Alport syndrome are also associated with PPCD. At the cellular level, the hexagonal corneal endothelial cells exhibit changes in cellular morphology and size [3]. Additionally, the affected corneal endothelial cells exhibit epithelial cell-like characteristics such as stratification, desmosomes, and microvilli [4]. In some cases, these abnormal cells can affect the iridocorneal angle and trabecular meshwork, leading to glaucoma. PPCD also exhibits genetic locus heterogeneity with linkage demonstrated to two different genomic loci: PPCD1 (MIM ID **#**122000), associated with an unknown variant on chromosome 20p11.2-q11.2, and PPCD3 (MIM ID **#**609141), associated with truncating mutations in the zinc finger E box-binding homeobox 1 gene (*ZEB1*) gene on chromosome 10p11.22.

**Fig 1:**
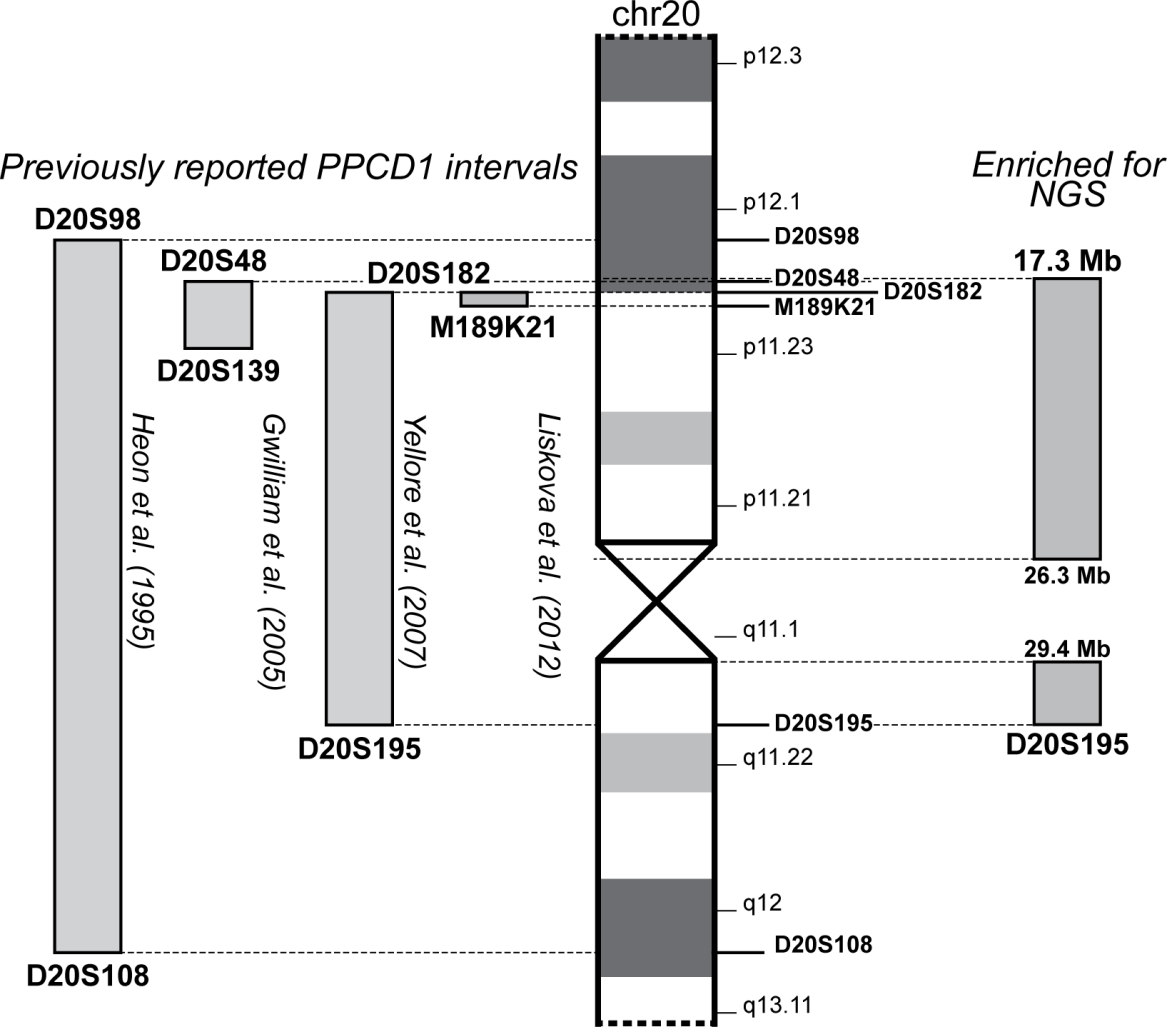
Abbreviated ideogram of chromosome 20 with PPCD1-associated intervals. Relative position of previously reported intervals associated with PPCD1 are on the left of the ideogram. Relative position of the interval enriched for NGS in this study is on the right of the ideogram. Ideogram and genomic coordinates are based on the hg19 reference build. *The interval reported by Hosseini et al. refines the original interval reported by Heon et al.

Multiple groups have reported PPCD1 families linked to a common region on chromosome 20, but the genetic basis for PPCD1 is still unknown (Fig 1). The first report for PPCD1 described genetic linkage to a locus on chromosome 20 between STS markers D20S98 and D20S108 [5]. Since then, multiple groups have reported other PPCD1 families, all showing linkage within the initial interval reported by Heon et al. [6–9]. All together, these studies suggest that the genetic basis for PPCD1 is found within the common support interval between D20S182 and D20S139 (approximately 3.6 cM or 1.8 Mb), which contains 32 genes according to the NCBI Annotation Release 105.

Despite these reports that map PPCD1 to a common locus on chromosome 20, screening of the coding regions of multiple candidate genes within the initial PPCD1 interval and the PPCD1 common support interval have failed to identify a causal variant [6–8, 10–14]. Since screening of the exon and exon-intron boundaries of the genes in the common support interval, and other genes outside of the common region, have failed to identify the causal variant for PPCD1, we used a targeted next-generation sequencing (NGS) approach to screen variants within the linked region bordered by flanking markers D20S182-D20S195 in a previously reported PPCD1 family [9]. We previously published a limited study showing the utility of targeted NGS for PPCD1 but herein describe a substantially more comprehensive and robust NGS approach for the identification of the genetic basis of PPCD1 [13].

## Materials and Methods

This study followed the Declaration of Helsinki and was approved by the Institutional Review Board at the University of California at Los Angeles (UCLA IRB# 94-07-243-(14-33A), 02-10-092-(4,11)). Written consent was obtained from all subjects in this study.

### Subject selection and DNA collection

A total of 29 members from an affected family, previously mapped to the PPCD1 locus on chromosome 20, were enrolled in this study [9]. Clinical characterization was previously described. Genomic DNA was purified from peripheral blood leukocytes using the FlexiGene DNA Isolation Kit (Qiagen, Valencia, CA) or extracted from buccal epithelial cells using the Oragene Saliva Collection Kit (DNA Genotek, Ottawa, Canada) according to the manufacturer’s instructions.

### Library preparation and next-generation sequencing

DNA from four affected and four unaffected members of the family previously mapped to the chromosome 20 locus were prepared for next-generation high-throughput sequencing at the UCLA Clinical Microarray Core. A DNA library was prepared using the Seqcap EZ Choice XL kit (Roche Diagnositics, Indianapolis, IN). In order to completely encompass the PPCD1 common support interval, a custom-designed oligonucleotide probe array (Roche NimbleGen Iceland LLc., Reykjavik, Iceland) was used to enrich for the previously linked chromosome 20 region in this family and an additional 500 Kbp 5’ of the linked interval (hg19: 17.3 Mbp– 31.8 Mbp). Highly repetitive regions such as the centromere were excluded from enrichment. High-throughput sequencing was performed on the Illumina HiSeq2000 platform (Illumina, Inc., San Diego, CA).

### Variant calling bioinformatics pipelines

FASTQ files were downloaded from the UCLA Clinical Microarray Core and processed with two independent bioinformatics pipelines.

#### Burrows Wheeler Aligner/GATK HaplotypeCaller pipeline (BWA/GATK)

All reference files for this pipeline were obtained from the Broad Institute’s reference file directory (https://www.broadinstitute.org/gatk/download/) associated with UCSC’s hg19 human reference genome. Files were then processed and analyzed according to recommendations from the Genome Analysis Toolkit (GATK) Best Practices Pipeline [15–17]. FASTQ files were first aligned to the hg19 reference genome with the Burrows Wheeler Aligner (BWA, http://bio-bwa.sourceforge.net/) for paired-end reads [18]. After alignment, Picard Tools was used to convert files from SAM to BAM, sort by genomic coordinates, and mark optical duplicates (http://broadinstitute.github.io/picard/). Files were then realigned to known indels using GATK. Variant calling for single-nucleotide variants (SNVs) and insertions/deletion (indels) was then conducted with GATK’s HaplotypeCaller algorithm.

#### BowTie2/SAMtools pipeline (BT2/SAM)

Using Partek® Flow® (Partek Inc., St. Louis, MI), FASTQ files were aligned to the hg19 human reference genome with BowTie2, and variant calling for SNVs and indels were performed with SAMtools using default settings.

### Variant filtering and annotation

Variant output files from both bioinformatics pipelines were analyzed using Partek® Genomics Suite® platform. The following criteria were utilized to filter candidate variants: (1) within the enriched interval (chr20: 17,316,434 bp– 26,319,280 bp and 29,420,138 bp– 31,826,081 bp); (2) read depth of ≥ 5; (3) quality score ≥ 20; (4) heterozygous, (allele frequency from 0.4–0.7); (5) not present in unrelated, unaffected controls; (6) present in affected family members and absent in unaffected family members; and (7) rare or novel (minor allele frequency (MAF) ≤ 0.05). MAFs were assigned using the Database of Single Nucleotide Polymorphisms (National Center for Biotechnology Information, National Library of Medicine; Bethesda, MD [dbSNP Build ID: 138]), and novel variants were defined as lacking a reference SNP cluster ID. After filtering, SNVs were then annotated with the Ensembl (v75) and RefSeq (compiled 02-02-2015 by Partek Inc.) annotation databases. SNVs were categorized as missense, nonsense, synonymous, 5’ or 3’ splice site, 5’ or 3’ untranslated region (UTR), promoter, intronic, or non-coding RNA (ncRNA). Indels were annotated with only the Ensembl (v75) database using Ensembl’s Variant Effect Predictor tool (VEP, http://grch37.ensembl.org/info/docs/tools/vep/index.html).

### SNV validation and screening of additional family members

Sanger sequencing was performed to validate the filtered NGS-detected SNVs. Validated SNVs located in protein-coding genes were screened for in an additional 19 family members (seven affected and 12 unaffected). Novel SNVs in protein-coding genes were screened for in 100 ethnically matched controls. Sequencing primers are given in S1 Table.

### *In silico* analysis of filtered promoter region SNVs and synonymous substitutions identified in protein-coding genes

Variants identified within the promoter regions of protein-coding genes were analyzed using JASPAR (http://jaspar.genereg.net/) in order to detect possible changes within transcription factor binding sites [19]. A threshold cutoff score of ≥ 75% was used. In addition, cryptic splice site prediction for synonymous SNVs was performed using MutPred Splice (http://mutdb.org/mutpredsplice/submit.htm) and NetGene2 (http://www.cbs.dtu.dk/services/NetGene2) [20, 21].

### Determination of corneal endothelial expression of protein-coding genes in which validated, filtered, SNVs were identified

Transcript levels of *OVOL2*, *CCM2L* and *THBD* in the corneal endothelium were previously determined by RNA-seq, while the level of the encoded proteins was determined by fluorescence-immunohistochemistry (F-IHC) [22]. A cadaveric donor cornea from an unaffected individual and two corneas from individuals with PPCD without a *ZEB1* mutation (non-PPCD3) obtained at time of surgery were fixed in 10% Tris-buffered formalin and subsequently paraffin embedded. Immunodetection was performed using a standard immunohistochemistry protocol with antibodies directed against OVOL2, CCM2L and THBD (S2 Table). In brief, sections were deparaffinized in Histo-Clear (National Diagnostics, Atlanta, GA) and rehydrated through a series of alcohols (100%, 95% and 80%) and water. Antigen retrieval was performed using proteinase-K digestion at 37°C for 15 minutes and sections were washed in PBST (PBS and 0.5% Tween 20). Non-specific epitope blocking was achieved by a 1 hour incubation with PBST supplemented with 1% bovine serum albumin and 10% normal serum. The sections were subsequently incubated overnight with each primary antibody diluted 1:500 (OVOL2) or 1:100 (CCM2L and THBD) in blocking buffer, followed by three washes in PBST. Incubation with a secondary antibody, Alexa Fluor 594 (Life Technologies, Carlsbad, CA), diluted 1:500 in blocking buffer was performed. After washing three times with PBST and one time with PBS, sections were mounted with Vectashield aqueous mounting medium containing 4′,6-diamidino-2-phenylindole (Vector Laboratories Inc., Burlingame, CA). To account for non-specific fluorescence, a control was performed using only the secondary antibody. Images were obtained using a fluorescence confocal microscope. Quantification of the fluorescence signal corresponding to each protein was performed using the Volocity 3D Image Analysis Software (PerkinElmer, Waltham, MA). Final fluorescence quantities were determined by subtracting non-specific fluorescence values, which were obtained by measuring the fluorescence in a field devoid of tissue in each image from the fluorescence in the endothelium of the secondary-only control.

### Copy number variant analysis using high-resolution array comparative genomic hybridization

Copy number variation (CNV) analysis was performed using genomic DNA samples from the aforementioned four affected and four unaffected individuals that underwent NGS. The genomic DNA samples were submitted to the UCLA Clinical Microarray Core for array comparative genome hybridization (aCGH) using a custom Agilent 8x60K array (Agilent Technologies, Inc., Santa Clara, CA). Interrogation of a 16.7 Mbp region (hg19: 17.3 Mbp– 34.0 Mbp) encompassing the linked PPCD1 locus and approximately 2.7 Mbps (0.54 Mbp 5’ and 2.17 Mbp 3’) of sequence flanking the PPCD1 locus was performed using 52,828 oligonucleotide probes. This design resulted in a median probe spacing of 159 bp. Data analysis was performed using the Agilent CytoGenomics 3.0 software. The raw data files are available from the GEO DataSets database (accession number GSE72617; National Center for Biotechnology Information [NCBI], Bethesda, MD, USA). CNV validation was performed using agarose gel electrophoresis of PCR products amplified with primers flanking the putative CNV (S1 Table).

## Results

### Sequencing reads align to the enriched region

**Fig 2:**
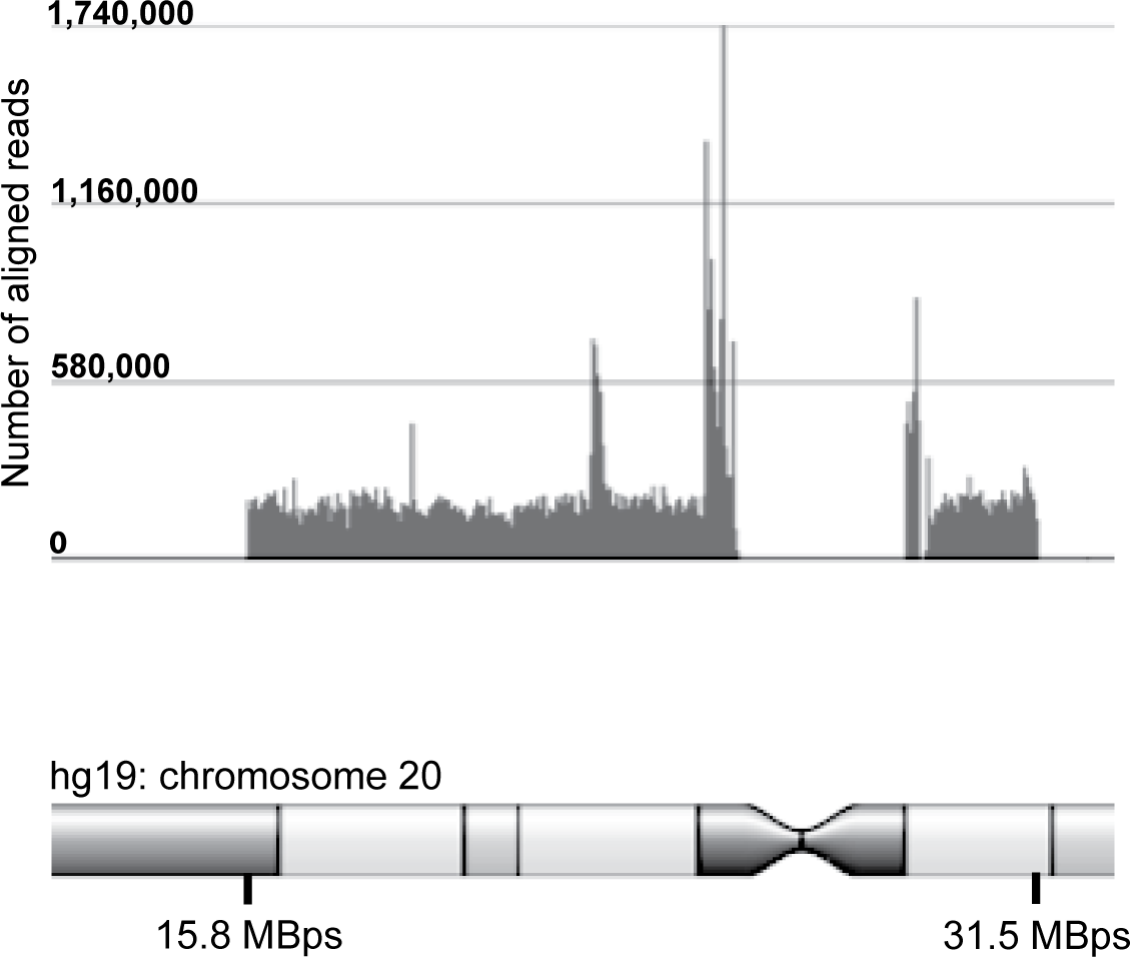
Coverage and read-depth of next-generation sequencing reads of the PPCD1 locus. Histogram depicts the number of reads aligning to the PPCD1 candidate region on chromosome 20 for a representative individual (hg19 reference sequence; histogram produced using Partek® Genomics Suite®).

After performing alignment algorithms using two bioinformatics pipelines, NGS reads from one representative sample that underwent NGS were confirmed to align to the enriched region on chromosome 20 (Fig 2). Oligonucleotide probes for regions of low complexity (e.g., centromere) were avoided and therefore lack reads.

### Comparison of two bioinformatics pipelines for the detection of SNVs

After variant filtering, a comparison of SNVs detected by the BT2/SAM and BWA/GATK pipelines was conducted to determine concordance between the two bioinformatics pipelines. The BT2/SAM pipeline detected a total of 839 SNVs and the BWA/GATK pipeline detected a total of 885 SNVs. Of these, 820 SNVs were concordant between both pipelines, while 19 SNVs were detected by only BT2/SAM and 65 SNVs were detected by only BWA/GATK.

### Comparison of two gene annotation databases for the classification of identified SNVs

**Fig 3:**
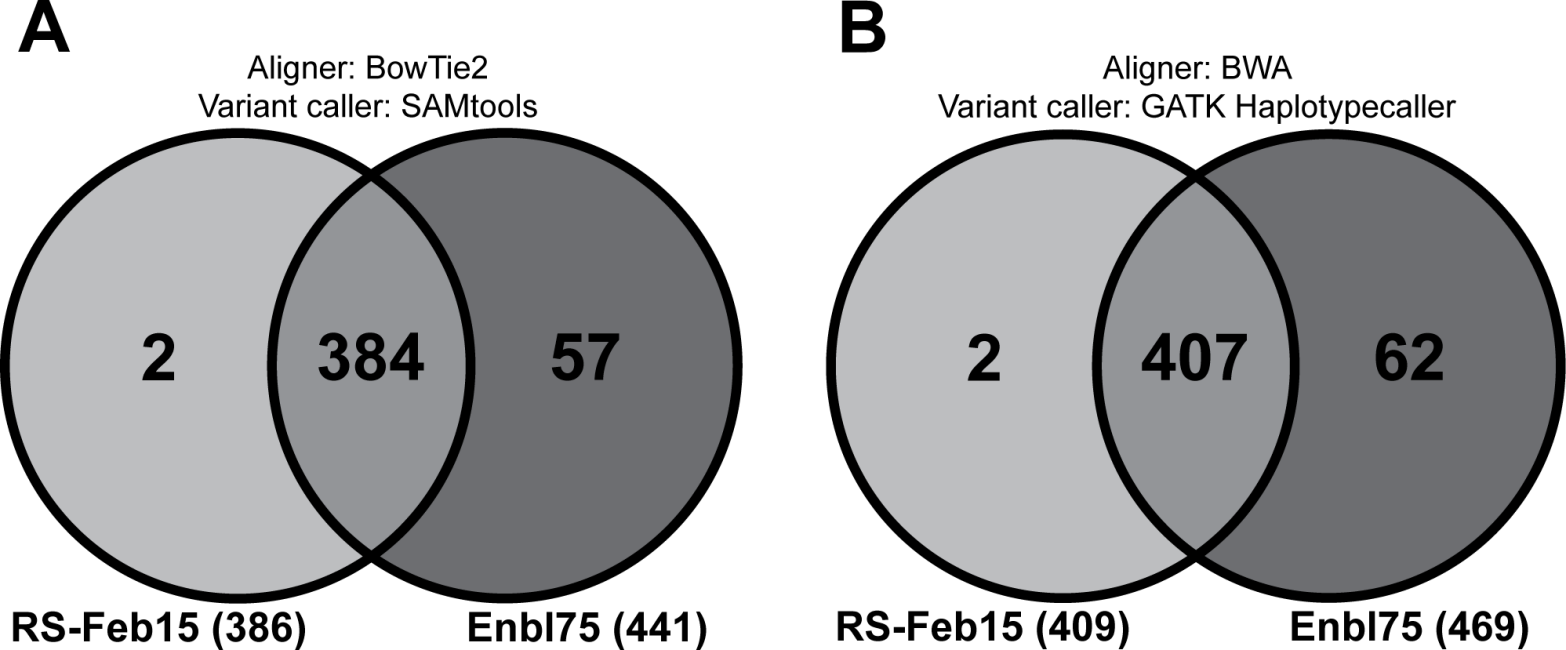
SNV annotation differs between annotation databases. Comparison of functional annotations between the RefSeq (RS-Feb15) and the Ensembl (Enbl75) databases for SNVs detected by both bioinformatics pipelines. (A) Number of annotated SNVs detected by the BowTie2-SAMtools pipeline. (B) Number of annotated SNVs detected by the BWA-GATK HaplotypeCaller pipeline.

After performing variant filtering, a comparison of annotated SNVs was conducted to determine concordance between annotation databases for each bioinformatics pipeline. For the BT2/SAM pipeline, 386 SNVs were annotated by the RefSeq database, 441 SNVs were annotated by the Ensembl database, and 384 annotated variants were concordant between both annotation databases (Fig 3A). For the BWA/GATK pipeline, 409 SNVs were annotated by the RefSeq database, 469 SNVs were annotated by the Ensembl database, and 407 SNVs were concordant between both annotation databases (Fig 3B).

### SNV and indel variant analysis of the PPCD1 interval

#### SNV analysis

The majority of detected SNVs were intergenic SNVs (not annotated) or annotated as intronic or ncRNA SNVs (data not shown). A total of 12 SNVs located in 10 genes that were neither intronic nor ncRNA passed our filtering criteria (Table 1). Three of the 10 genes were protein-coding: *OVOL2*, *THBD* and *CCM2L*. Two of the 12 SNVs were coding region variants, resulting in synonymous substitutions in *OVOL2* and *CCM2L*. Nine of the 12 SNVs were located in the promoter regions of eight genes, including two variants in the promoter region of *OVOL2*. The remaining SNV was located in the 3’ untranslated region (UTR) of *THBD*. Of the two SNVs that were novel, only one, NM_021220:c.-307T>C in *OVOL2*, affected a protein-coding transcript.

**Table 1:** Annotated candidate SNVs in the PPCD1 interval.

Position	Ref	Alt	Gene symbol	RS number	MAF	Functional annotation	Exon	HGVS nucleotide change	RefSeq ID	Ensembl ID	Transcript biotype^†^
18,022,362	C	A	*OVOL2*	rs6111803	0.0403	Synonymous	3	c.327C>A	NM_021220	ENST00000278780	Protein-coding
18,031,455	A	T	*OVOL2*	rs6045164	0.0409	Promoter	-	n.-72A>T	-	ENST00000462208	Processed transcript
18,038,585	T	C	*OVOL2*	Novel	-	Promoter	-	c.-307T>C	NM_021220	ENST00000278780	Protein-coding
20,835,294	C	T	*MRPS11P1*	rs540742459	0.0016	Promoter	-	n.-9C>T	-	ENST00000437558	Processed pseudogene
21,206,234	A	G	*KIZ*	rs759099707	n/a	Promoter	-	n.-544A>G	-	ENST00000441136	Processed transcript
23,028,063	A	G	*THBD*	rs11696919	0.0192	3' UTR	-	c.*351A>G	NM_000361	ENST00000377103	Protein-coding
23,564,038	G	A	*RP11-218C14*.*2*	rs186922449	0.005	Promoter	-	n.-244G>A	-	ENST00000437612	Unprocessed pseudogene
25,636,109	G	A	*RN7SL594P*	rs145320819	0.0032	Promoter	-	n.-592G>A	-	ENST00000470590	miscRNA
26,045,831*	C	T	*FAM182A*	Novel	-	Promoter	-	n.-452C>T	-	ENST00000439881	lincRNA
26,175,891*	C	T	*MIR663A*	rs145247716	0.0014	Promoter	-	n.-493C>T	-	ENST00000596717	lincRNA
29,638,726	G	C	*MLLT10P1*	rs567212483	0.0268	Promoter	-	n.-666G>C	NR_045115	ENST00000418346	Processed pseudogene
30,616,835	G	A	*CCM2L*	rs6089151	0.0188	Synonymous	7	c.1107G>A	NM_080625	ENST00000262659	Protein-coding

* Indicates variants detected by only BWA/GATK

^†^Transcript biotype was determined from only the Ensembl database

Ref: wild-type, reference allele; Alt: alternative, mutant allele. Intergenic, intronic, and ncRNA SNVs were excluded. Each variant is transcript specific.

#### Indel analysis

Since indel realignment is not available in Partek® Flow®, only indels detected by the BWA/GATK pipeline were analyzed to determine segregation with the affected phenotype. Due to difficulties in indel annotation in Partek® Genomics Suite®, Ensembl’s VEP tool was utilized to annotate indels. A total of 168 indels segregated with the affected status, with 159 indels annotated as intergenic or intronic. The remaining nine indels were located in seven genes, all of which were protein-coding (Table 2). Validation of these nine indels was not performed since the majority of the indels are located in regions of low complexity. Five of the nine indels were insertions located in intron 1 of *CRNKL1* and the promoter of *C20orf26* while the other four indels were located in the non-coding regions of five different genes. One of these four indels, which was mapped to the intronic region of *HCK*, was also mapped to the exonic region of a non-protein-coding transcript for *HCK* within the Ensembl database.

**Table 2:** Annotated candidate indels in the PPCD1 interval.

Position	Indel type	Gene symbol	Reference allele	Alternative allele	Functional annotation	RS Number	MAF	RefSeq ID	Ensembl ID
18,469,617	Insertion	*RBBP9*	G	GTGTGTGTGTA	3' UTR	rs35822681	N/A	NM_006606	ENST00000337227
20,034,644^†^	Insertion	*CRNKL1*	G	GTT	Intron 1	rs772688050	N/A	NM_016652	ENST00000377340
20,034,644^†^	Insertion	*C20orf26*	G	GTT	Promoter	rs772688050	N/A	NM_015585	ENST00000245957
20,034,653^†^	Insertion	*CRNKL1*	G	GT	Intron 1	Novel	-	NM_016652	ENST00000377340
20,034,653^†^	Insertion	*C20orf26*	G	GT	Promoter	Novel	-	NM_015585	ENST00000245957
20,034,661^†^	Insertion	*CRNKL1*	T	TG	Intron 1	rs761128007	N/A	NM_016652	ENST00000377340
20,034,661^†^	Insertion	*C20orf26*	T	TG	Promoter	rs761128007	N/A	NM_015585	ENST00000245957
20,034,665^†^	Insertion	*CRNKL1*	T	TTGTTTTTG	Intron 1	rs776918983	N/A	NM_016652	ENST00000377340
20,034,665^†^	Insertion	*C20orf26*	T	TTGTTTTTG	Promoter	rs776918983	N/A	NM_015585	ENST00000245957
20,035,056^†^	Insertion	*CRNKL1*	T	TACACACAC	Intron 1	rs759860912	N/A	NM_016652	ENST00000377340
20,035,056^†^	Insertion	*C20orf26*	T	TACACACAC	Promoter	rs759860912	N/A	NM_015585	ENST00000245957
30,434,096	Insertion	*FOXS1*	G	GACGACAC	Promoter	rs144761785	N/A	NM_004118	ENST00000375978
30,461,617^†^	Deletion	*TTLL9*	GAAGGAAGGAAGA	G	Intron 1	Novel	-	NM_001008409	ENST00000535842
30,461,617^†^	Deletion	*DUSP15*	GAAGGAAGGAAGA	G	Promoter	Novel	-	NM_080611	ENST00000339738
30,660,366^†^	Deletion	*HCK*	TTAAAAAAAA	T	Intron 2	rs3838038	N/A	NM_002110	ENST00000520553
30,660,366^†^	Deletion	*HCK*	TTAAAAAAAA	T	Exon 2*	rs3838038	N/A	-	ENST00000470092

*ENST00000470092 retains an intron and produces no protein product.

^†^Indels that have multiple annotations.

All transcripts except ENST00000470092 are protein-coding.

### SNV validation and screening of additional family members

Of the 12 SNVs that survived the filtering criteria, 11 SNVs were confirmed by Sanger sequencing to be present in the four affected individuals who underwent NGS (the SNV in *FAM182A* (n.-452C>T) was not detected) and each was confirmed to be absent in the four unaffected individuals who underwent NGS (S1 Fig). Four of the 12 SNVs (*OVOL2* c.327C>A; *OVOL2* c.-307T>C; *THBD* c.351A>G; *CCM2L* c.1107G>A) were located in protein-coding genes. Nineteen additional family members (7 affected and 12 unaffected) who did not undergo NGS were screened for each of these four SNVs. Three of the four SNVs (*OVOL2* c.327C>A, *OVOL2* c.-307T>C, and *THBD* c.351A>G) segregated with the affected status of the additional family members. Although present in all affected family members, *CCM2L* c.1107G>A was also identified in one unaffected family member. *OVOL2* c.-307T>C, the only novel SNV found within a protein-coding gene, was not found in 100 controls.

### *In silico* analysis of promoter region SNVs and synonymous substitutions identified in protein-coding genes

Of the variants that passed the filtering criteria, *OVOL2* c.-307T>C was the only variant found within the promoter region of a protein-coding gene. Thus, this variant was analyzed to determine whether it could cause any changes to the transcription factor binding sites within the *OVOL2* promoter. According to JASPER, *OVOL2* c.-307T>C is predicted to cause the formation of an additional FOXO3 enhancer transcription factor binding site (with a relative score of 77.8%) to the *OVOL2* promoter.

Of the other variants that passed the filtering criteria, *OVOL2* c.327C>A and *CCM2L* c.1107G>A were annotated as synonymous substitutions. Thus these variants were analyzed with MutPred Splice and NetGene2 to determine whether a cryptic splice site would be created. MutPred Splice predicted that the synonymous substitution in exon 3 of *OVOL2* (c.327C>A) was a splice neutral variant with a score of 0.17. Additionally, NetGene2 did not predict that this synonymous substitution in *OVOL2* would alter splicing, predicting the wild type splice acceptor and splice donor sites that flank exon 3 with confidence values of 1.00. MutPred Splice predicted that the synonymous substitution in exon 7 of *CCM2L* (c.1107G>C) was also a splice neutral variant with a score of 0.09 while NetGene2 predicted the creation of a splice acceptor site with a confidence of 0.19. The wild type splice acceptor site flanking exon 7 of *CCM2L* was predicted with a confidence value of 0.33, and the wild type splice donor site was predicted with a confidence value of 0.95.

### Expression of *OVOL2*, *CCM2L* and *THBD* in PPCD corneal endothelium

**Fig 4:**
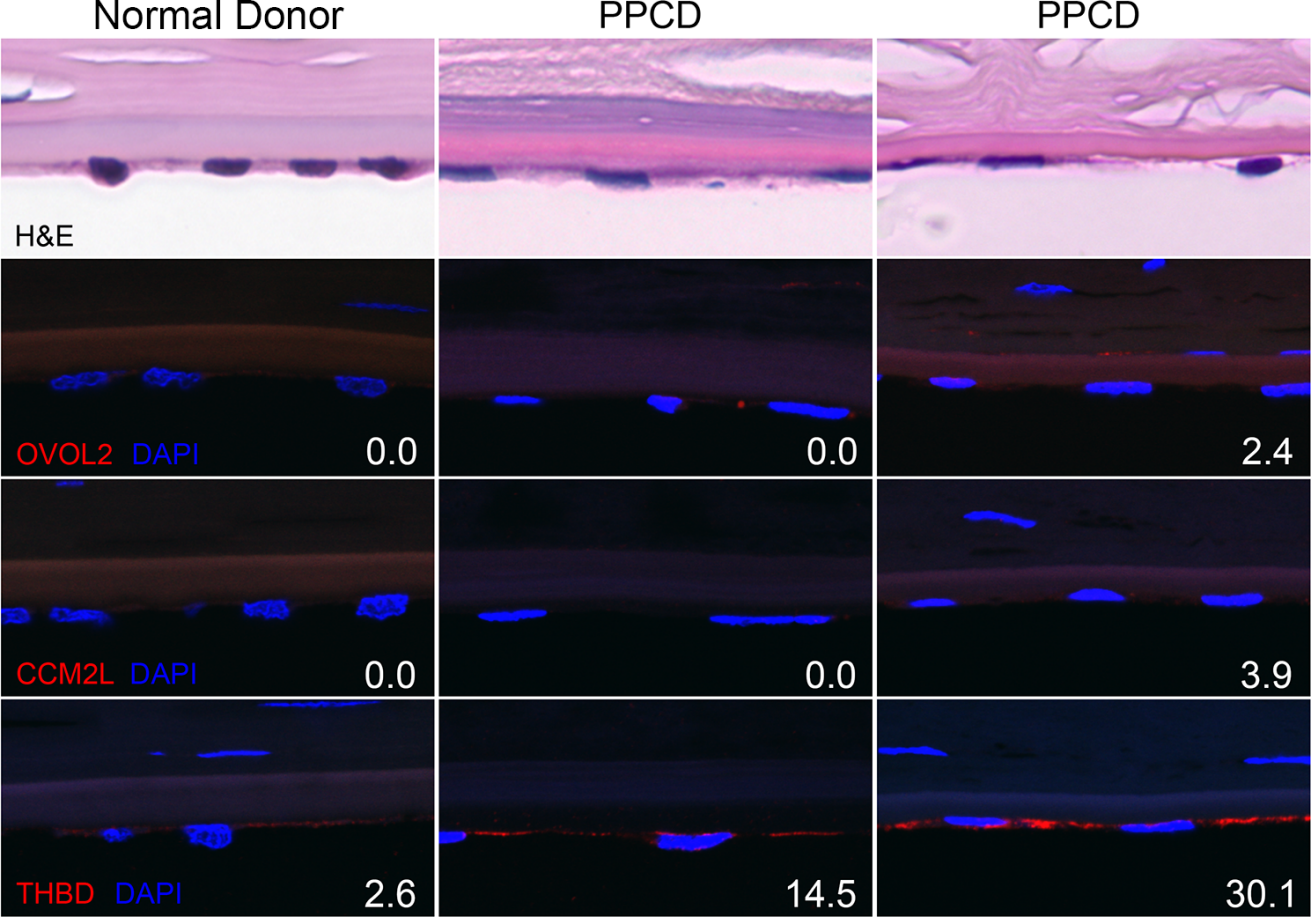
Detection of OVOL2, CCM2L and THBD in normal donor and PPCD corneal endothelium by F-IHC. H&E: Hematoxylin and eosin stain (row 1). Primary antibodies directed against the proteins encoded by *OVOL2* (row 2), *CCM2L* (row 3) and *THBD* (row 4) were used to detect protein expression in the corneal endothelium of a normal donor (column 1) and two PPCD corneas (columns 2 and 3). A secondary antibody conjugated to a fluorescent moiety (Alexa Fluor 594, red) was used to visualize the localization of the primary antibodies. The sections were counterstained with DAPI, which stained the nuclei blue. Numbers located at lower right corner of each panel represent the quantification of the fluorescent signal in fluorescence units per pixel (FU/px), corrected for autofluorescence.

A recent study using RNA-seq to profile the *ex vivo* human corneal endothelial cell transcriptome demonstrated transcript levels for *OVOL2* (0.03 RPKM) and *CCM2L* (0.00 RPKM) at levels significantly below the background cutoff of 1 RPKM, while THBD (50.87 RPKM) was significantly above this cutoff [22]. Expression of the proteins encoded by *OVOL2*, *CCM2L* and *THBD* was investigated using F-IHC (Fig 4). In agreement with the transcript levels determined by RNA-seq, THBD was detected in the normal donor endothelium while OVOL2 and CCM2L were not. Similarly, one of the PPCD corneas did not show expression of OVOL2 or CCM2L, while the second PPCD cornea demonstrated increased OVOL2 (2.4 fluorescence units per pixel (FU/px)) and CCM2L (3.9 FU/px) expression compared with the normal donor cornea. THBD was detected in both normal donor (2.6 FU/px) and PPCD corneas (14.5 and 30.1 FU/px) with a marked increase in both PPCD corneas compared with normal donor cornea.

### Copy number variant analysis of the PPCD1 interval

CNV analysis was performed to identify a potentially pathogenic microdeletion or microinsertion in the PPCD1 interval. DNA samples from the four affected and the four unaffected individuals for whom NGS was performed were subjected to high-resolution aCGH. Fourteen CNVs (7 gains and 7 losses) were identified in at least one individual and ranged in size from 121 to 413,066 base pairs. A single CNV (318 bp loss) within an intron of *OVOL2* was identified in all the affected individuals but none of the unaffected individuals. However, subsequent validation by PCR did not identify the 318 bp loss in the region predicted by aCGH (data not shown).

## Discussion

In this study, we present, to the best of our knowledge, the first screening of the PPCD1 interval to identify potentially pathogenic coding and non-coding SNVs, indels and CNVs. NGS was used to sequence the entire linked region, which includes the PPCD1 common support interval, excluding regions of low complexity such as the centromere. With adequate coverage of the sequenced region, two independent bioinformatics pipelines were utilized for alignment and variant calling, and two independent annotation databases were utilized to annotate SNVs. Indels were analyzed and annotated using only the BWA/GATK pipeline and the Ensembl annotation database. After performing genetic filtering, a total of 11 validated candidate SNVs in nine genes and nine non-validated indels in seven genes were identified. Additionally, aCGH was used to interrogate CNVs in the linked region, although the single CNV identified within the PPCD1 interval proved not to be present after validation was performed. The most plausible explanation for the identification of a CNV that is not subsequently observed by PCR is a false positive result associated with the phenomenon of competitive hybridization observed with high-density array designs. In this case, the observation of the 318 bp loss only in the affected individuals may be due to the presence of a smaller genetic variation (i.e., SNV or small indel) in these individuals that results in an alteration in the competitive hybridization of the three involved probes.

No non-synonymous coding variants were detected in any of the annotated genes within the linked region, consistent with previous reports that failed to identify non-synonymous coding region variants in multiple genes within the PPCD1 locus. However, the recent association of another corneal dystrophy with a synonymous substitution in *COL17A1* that creates a cryptic splice donor site, resulting in the loss of 18 amino acids, highlights the potential pathogenicity of synonymous substitutions [23, 24]. Therefore, we performed an *in silico* analysis of the synonymous substitutions identified in *OVOL2* and *CCM2L*. The synonymous substitutions in *OVOL2* was not predicted to create a cryptic splice site while one bioinformatics tool predicted that the synonymous substitution in *CCM2L* creates a relatively weak cryptic splice acceptor site in comparison to the wild type splice acceptor site. Thus, the exclusion of potentially pathogenic coding region SNVs and CNVs in the PPCD1 interval indicates that the genetic variant responsible for PPCD1 is likely in a non-coding region. Of particular interest, the novel SNV that we report in the promoter region of *OVOL2* (c.-307T>C) results in the formation of a binding site motif for the transcription factor FOXO3, a promoter of gene transcription [25]. Given the pathogenic role of *ZEB1* in PPCD3, and the fact that OVOL proteins are involved in the suppression of *ZEB1* transcription, *OVOL2* is a functional as well as positional candidate gene for PPCD1 [26–29]. To this end, the observation that OVOL2 protein was elevated in the corneal endothelium of one of the corneas from two individuals with PPCD provides some evidence to support this hypothesis. Although these corneas were obtained from individuals in whom Sanger sequencing of the exons in the *ZEB1* gene did not reveal a pathogenic mutation, it may not necessarily be assumed that both of these individuals have PPCD1 as neither are members of a family linked to the PPCD1 interval. Thus, the failure to detect *OVOL2* expression in the corneal endothelium of one individual does not exclude the possibility that OVOL2 may be ectopically expressed in the corneal endothelium in individuals with PPCD1.

Although intronic, ncRNA, and intergenic variants were not analyzed in this study, these variants may have potentially pathogenic effects. In particular, intronic variants may also lead to formation of cryptic splice sites and aberrant alternative splicing. Additional regulatory elements may also exist in the intronic and intergenic regions, which may cause abnormal expression of protein-coding genes. Additionally, studies show that ncRNA such as microRNA and long intergenic non-coding RNA, have potentially important functional roles in regulating gene expression [30]. Since the causative variant or gene has yet to be identified, we advocate identification and screening of non-coding variants of the PPCD1 locus and suggest that variants affecting the expression of *OVOL2* may be causative of PPCD1.

## Supporting Information

S1 Table Primers for validation of detected variants. (DOCX)

S2 Table Antibodies used for fluorescence immunohistochemistry. (DOCX)

S1 Fig Validation of the 12 filtered SNVs detected by NGS. WT: wild-type sequence. MU: mutant sequence. (TIF)
