# Supplementary material for: Identification of Potentially Pathogenic Variants in the Posterior Polymorphous Corneal Dystrophy 1 Locus
Source: PLoS One. 2016 Jun 29;11(6):e0158467. doi: 10.1371/journal.pone.0158467 (PMC4927100; doi:10.1371/journal.pone.0158467)
Supplement: S1 Table — (DOCX) [file pone.0158467.s001.docx]

**S1 Table.** Primers for validation of detected variants

| **Primer Name** | **Sequence** | **Product Size** |
| --- | --- | --- |
| *OVOL2* c.327C>A Forward | TGCCAGTTGTTACTTCACCTT | 335 bp |
| *OVOL2* c.327C>A Reverse | CTTCAGGTCGAAGGTGTCGT |  |
| *OVOL2* n.-72A>T Forward | CATTTGGCCAACTGACTTCTC | 258 bp |
| *OVOL2* n.-72A>T Reverse | CCTTCATCAAGAATCCATTCC |  |
| *OVOL2* c.-307T>C Forward | AGGCTCCTCCTCTTCACCAG | 548 bp |
| *OVOL2* c.-307T>C Reverse | AGGCGAGCTCTTCGAGGTAA |  |
| *MRPS11P1* n.-9C>T Forward | CTTGACCACCAGGTAACAATG | 227 bp |
| *MRPS11P1* n.-9C>T Reverse | GCTACCATCTTCTCTTCAATG |  |
| *KIZ* n.-544A>G Forward | GTCAGGCTAGAAAGTGAAACC | 307 bp |
| *KIZ* n.-544A>G Reverse | CATGAGGTGTGTGGCAATTC |  |
| *THBD* c.351A>G Forward | AACGAAGACACAGACTGCGA | 382 bp |
| *THBD* c.351A>G Reverse | CAGGCACAGGTAGGGTGACT |  |
| *RP11-218C14.2* n.-244G>A Forward | CCTACTTGTGAATGAGAACATG | 374 bp |
| *RP11-218C14.2* n.-592G>A Reverse | GGAAAGAAGAGAGAGGAAAGG |  |
| *RN7SL594P* n.-592G>A Forward | CTTGTACAATAGGCATGTACC | 822 bp |
| *RN7SL594P* n.-592G>A Reverse | CACCAGAGCTCCCAAGTATAT |  |
| *FAM182A* n.-452C>T Forward | GGTTCTGCAGATAACTGGCG | 231 bp |
| *FAM182A* n.-452C>T Reverse | CCAGATACTCTGATATGCATGA |  |
| *MIR633A* n.-493C>T Forward | CTGCACAGCAAACACTCAAC | 434 bp |
| *MIR633A* n.-493C>T Reverse | GGAGGATTGTGGAGTCATACA |  |
| *MLLT10P1* n.-666G>C Forward | CCAGTTATTACATTGAGGACAAAG | 330 bp |
| *MLLT10P1* n.-666G>C Reverse | CCTGGCATCAGAGGGAGAC |  |
| *CCM2L* c.1107G>A Forward | CAGCATGCAGTCTAGGATGA | 341 bp |
| *CCM2L* c.1107G>A Reverse | GACCTCTGATGTCTTCTGCTT |  |
| *OVOL2* Intron 3 Del Forward | TCCTGGACCTCCTGACTCCT | 1986 bp |
| *OVOL2* Intron 3 Del Reverse | CATGGAGACACACCTTCATCAG |  |
