# Supplementary material for: Identification of Potentially Pathogenic Variants in the Posterior Polymorphous Corneal Dystrophy 1 Locus
Source: PLoS One. 2016 Jun 29;11(6):e0158467. doi: 10.1371/journal.pone.0158467 (PMC4927100; doi:10.1371/journal.pone.0158467)
Supplement: S2 Table — (DOCX) [file pone.0158467.s002.docx]

| Target Protein | Isotype* | Immunogen Species | Dilution | Vendor | Catalog Number | Registry ID^#^ |
| --- | --- | --- | --- | --- | --- | --- |
| Primary antibodies | | | | | | |
| CCM2L | rabbit IgG | human | 1:100 | Sigma-Aldrich | HPA008434 | AB_1845671 |
| OVOL2 | rabbit IgG | human | 1:500 | Abcam | Ab83265 | AB_1860989 |
| THBD | sheep IgG | human | 1:100 | R&D Systems | AF3947 | AB_1061682 |
| Secondary antibodies | | | | | | |
| Rb-IgG | donkey IgG (A594) | rabbit | 1:500 | Life Technologies | A-21207 | AB_10049744 |
| Gt-IgG | donkey IgG (A594) | goat | 1:500 | Life Technologies | A-11058 | AB_10563390 |

**S2 Table. Antibodies used for fluorescence immunohistochemistry.**

*All antibodies are polyclonal.

^#^Unique identifiers were obtained from <http://antibodyregistry.org/> (accessed October 15, 2015)
