# Supplementary figures and images for: Identification of Potentially Pathogenic Variants in the Posterior Polymorphous Corneal Dystrophy 1 Locus
Source: PLoS One. 2016 Jun 29;11(6):e0158467. doi: 10.1371/journal.pone.0158467 (PMC4927100; doi:10.1371/journal.pone.0158467)

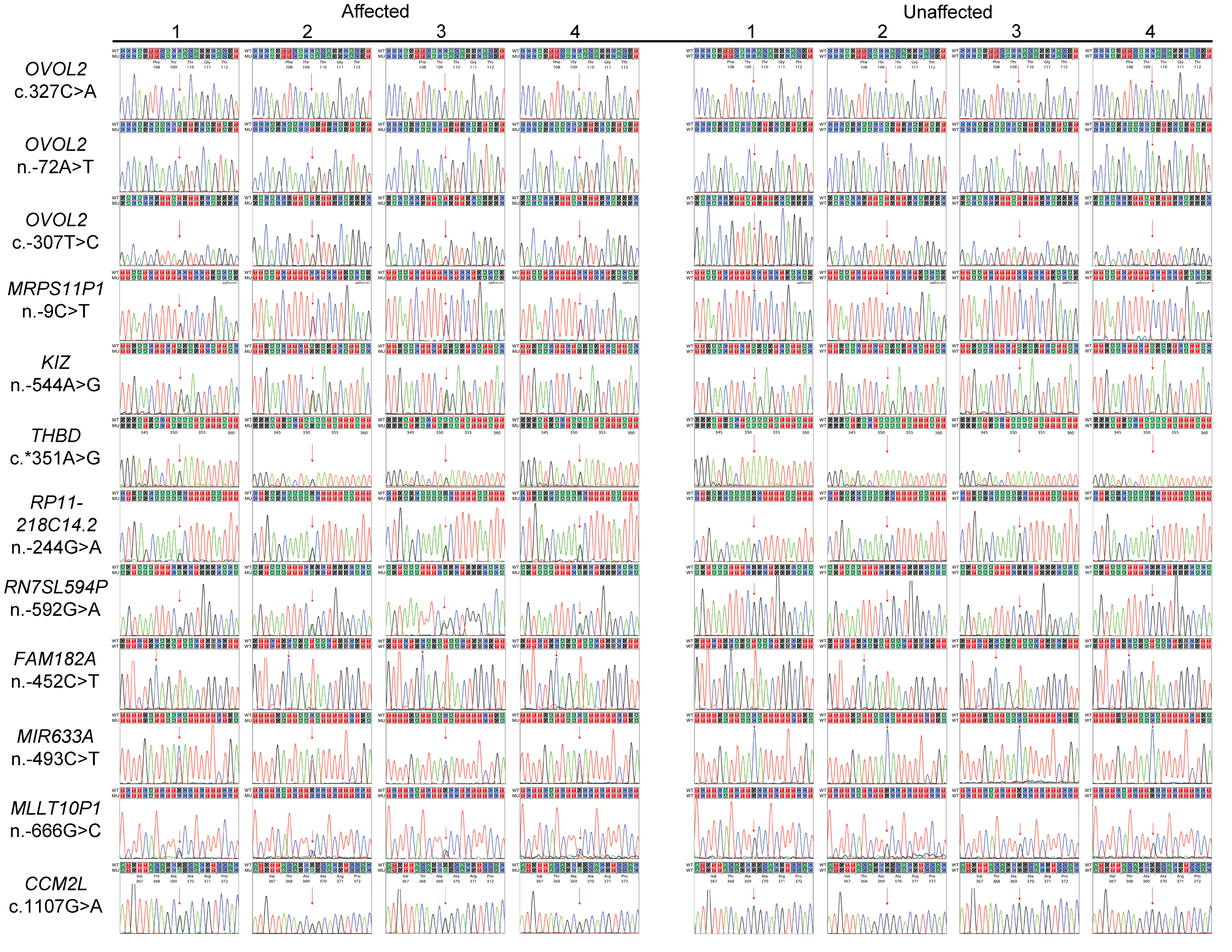

Supplement: S1 Fig — (TIF) [file pone.0158467.s003.tif]
